# Unexpected Ureter Within an Inguinal Hernia

**DOI:** 10.7759/cureus.36691

**Published:** 2023-03-26

**Authors:** Brodie D Laurie

**Affiliations:** 1 General Surgery, Sir Charles Gairdner Hospital, Perth, AUS

**Keywords:** hernia repair, general surgery, ureteroinguinal hernia, ureter, inguinal hernia

## Abstract

Inguinal hernias containing a ureter are a rare occurrence. They are rarely diagnosed pre-operatively and can lead to serious complications if inadvertently damaged during hernia repair. We present the case of a 36-year-old obese male who was found to have a ureter within his inguinal hernia intra-operatively. Due to imaging performed at another hospital, we have both pre and post-operative imaging demonstrating the ureter, its course into the inguinal hernia and its subsequent reduction back into the retroperitoneal space. We discuss the epidemiology of this phenomenon, the clinical implications and methods that have been suggested for pre-operative diagnosis.

## Introduction

Inguinal hernia repair is one of the most common surgical procedures performed worldwide [1]. It is generally a well-tolerated procedure with a low chance of significant complications. Finding a ureter within an inguinal hernia is a very rare occurrence, with less than 150 cases reported in the English literature [2-7]. Risk factors for ureteroinguinal hernia include male sex, age over 50, having a history of renal transplant and they are more common on the right side [7]. The presence of a ureter within an inguinal hernia may be suspected based on symptoms of ureteric obstruction such as dysuria or flank pain. Ultrasound may diagnose upstream hydronephrosis and cross-sectional imaging with CT can identify the ureter's course through the inguinal hernia. However, they are rarely diagnosed pre-operatively and if inadvertently damaged, can have life-long consequences for the patient [3,7]. In this paper, we present the case of a patient who underwent an open inguinal hernia repair where a ureter was found within his hernial contents, subsequently preserved and reduced back into the retroperitoneal space. We also review the literature around the topic, including proposed algorithms in pre-operative diagnosis of the ureteroinguinal hernia and how to avoid inadvertent damage to this vital structure.

## Case presentation

The patient was a 36-year-old obese male referred from a rural town for elective repair of bilateral inguinal hernias in a metropolitan hospital. He first noticed the hernias approximately three years prior but had not sought medical advice previously. They were causing him some intermittent, dull inguinal discomfort however his main complaint was his pronounced inguinoscrotal swelling. He reported no symptoms of ureteric or bowel obstruction. He had a body mass index (BMI) of 52, but aside from that he had no significant medical history and was a non-smoker. His only surgical history was an emergency laparoscopic appendicectomy one month prior at a different regional hospital. The appendix was noted to be gangrenous intra-operatively; however, the patient had an uncomplicated recovery and was discharged home. On examination, his vitals were all normal and he had bilateral, irreducible, non-tender inguinal hernias. His pre-operative blood tests, including renal function, were all normal and a routine urine dipstick showed no abnormalities. Due to the anesthetic protocol in place for the rural health service, he was unable to have an elective procedure at his local hospital due to a BMI greater than 50.

The decision was made for an open approach under general anesthesia in the setting of a deep abdominal pannus, the size of the hernias, and recent laparoscopic surgery potentially obliterating the plane required for laparoscopic repair. Following exposure of the right inguinal canal, a huge mass of fatty tissue was noted to be herniating through a very dilated (>6cm) deep inguinal ring which was causing distortion of the canal. This was mostly extraperitoneal fat with no obvious peritoneal sac found. Following careful dissection, the vas deferens and vessels were identified. However, an additional tubular structure 7mm in diameter was also found within the herniated tissues. Gentle stimulation provoked what appeared to be subtle vermiculation consistent with a ureter and subsequent intra-operative fine needle aspiration yielded some urine-like fluid. The ureter was reduced, most of the excess fat was resected and the Lichtenstein mesh repair of the hernia was otherwise uncomplicated. Given the unexpected findings, the decision was made to delay the repair of the left side until the anatomy could be verified with imaging.

**Figure 1:**
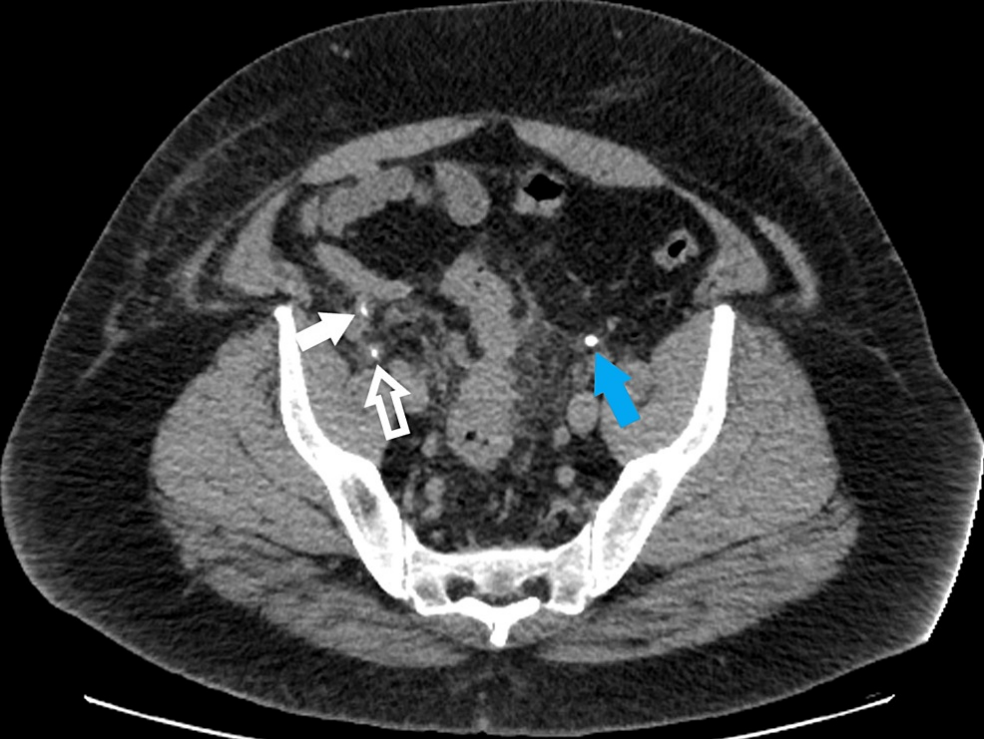
Post-operative CT Intravenous Pyelogram showing left ureter (blue arrow), proximal right ureter (solid white arrow) and distal right ureter (hollow white arrow).

The patient had an uneventful post-operative recovery and reported no issues at his follow-up appointment. A CT Intravenous Pyelogram was performed the following day to confirm anatomy, ensure no right-sided ureteric injury and plan for repair of the left-sided inguinal hernia. This CT confirmed that the right ureter had been reduced into the retroperitoneal space (Figure 1). As its course from the kidney to the bladder was now shortened, the ureter loops and both proximal and distal limbs can be seen. There was no obvious injury nor extravasation of contrast to suggest a urine leak.

 

**Figure 2:**
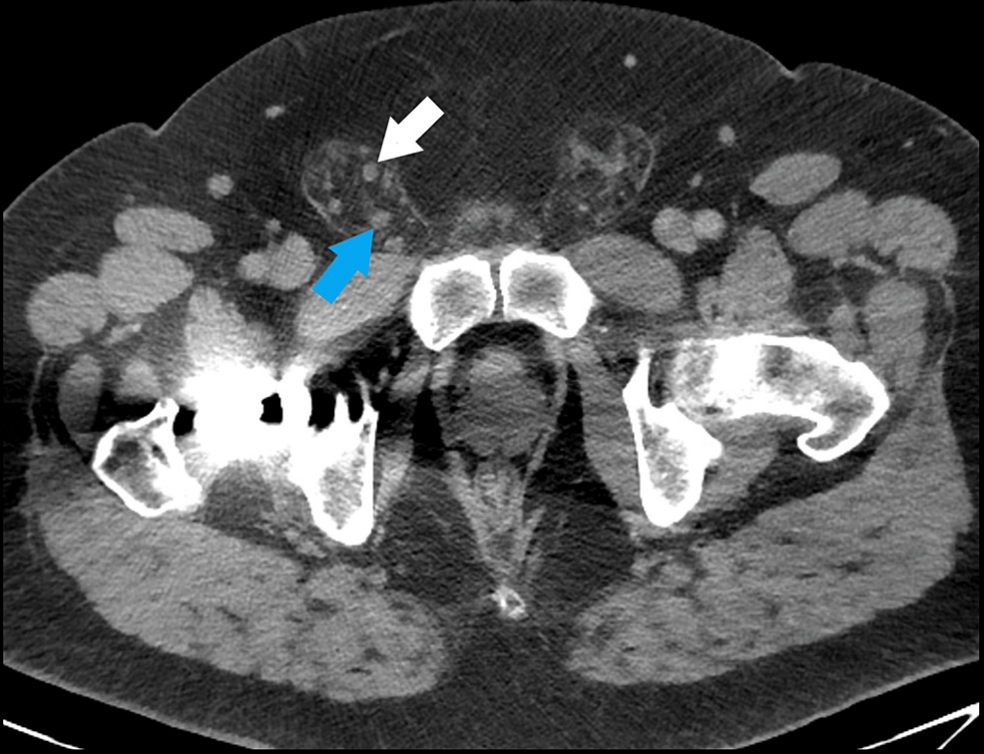
Pre-operative CT showing proximal right ureter (white arrow) and distal right ureter (blue arrow) within a right inguinal hernia.

**Figure 3:**
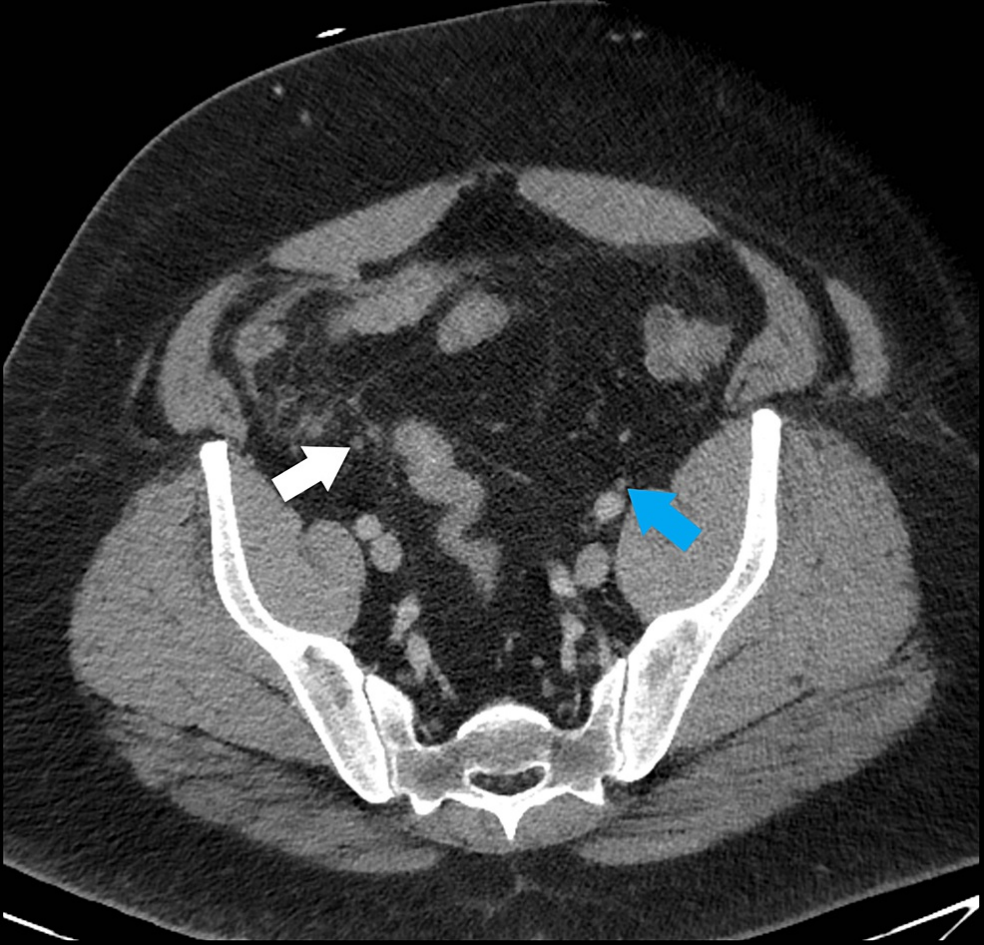
Pre-operative CT showing right ureter (white arrow) coursing far more anteriorly than left ureter (blue arrow).

Pre-operative imaging from the patient’s admission for appendicitis was requested from his treating hospital and was reviewed when available. This confirmed the presence of the right ureter looping within his right inguinal hernia at that time (Figure 2). Axial images at the level of the mid ureter show the left ureter just anterior to the iliac artery while the right ureter is coursing in a far more anterior position (Figure 3).

The patient had normal urea and creatinine levels post-operatively during his inpatient admission as well as at his follow-up appointment. He decided to defer the repair of his left inguinal hernia due to difficulties coordinating time off work, so this has yet to be carried out.

## Discussion

Ureteroinguinal hernias are rare in patients with native kidneys [8,9]. They are generally divided into paraperitoneal and extraperitoneal variants. Paraperitoneal-type ureteroinguinal hernias occur due to adhesion between the ureter and the herniating peritoneal sac. In contrast, extraperitoneal-type hernias are thought to be due to the congenital fusion between the ureter and the genitoinguinal ligaments [5,7]. The paraperitoneal type has previously been reported to account for 80% of ureteroinguinal hernias with extraperitoneal type making up the remaining 20%. However, much of the literature, including this case, are case reports featuring extraperitoneal hernias [2-3,5,7]. It is unclear whether this reflects an increased prevalence of this type of hernia or publication bias.

Ureteroinguinal hernias are more common on the right side, potentially due to the position of the fascia of Toldt on the left side, which provides a more consistent fixation in the retroperitoneum [7,10]. It is uncommon to diagnose them pre-operatively, which makes them prone to injury during surgical repair [3,7]. In this case, in hindsight, we had external imaging demonstrating the presence of a right-sided ureter within an inguinal hernia with mild ipsilateral hydronephrosis.

Algorithms for pre-operative diagnosis of a ureter containing an inguinal hernia have been suggested [7]. However, they rely on the patient to have either symptoms or biochemical evidence of ureteric obstruction - something which is not present in most cases. Patients do not typically have a renal tract ultrasound in preparation for inguinal hernia repair and cross-sectional imaging is not routinely required [11]. Given the low pre-test probability for this finding in most patients with inguinal hernias, routine imaging in an attempt to diagnose this is unlikely to be cost-effective or helpful. Risk factors that may help identify patients with a higher pre-test probability include obesity, male sex, and the presence of a large right-sided inguinoscrotal hernia [2,7].

As it is unlikely to be diagnosed pre-operatively, keeping the possibility of a ureter containing an inguinal hernia in mind when performing hernia repair is imperative. Any tubular structure with an atypical appearance for the vas deferens should be a red flag. Care should be taken prior to ligation of any large volume of subcutaneous tissue that is not clearly within a peritoneal sac, in case it may be retroperitoneal fat containing an unexpected ureter.

## Conclusions

A ureter within an inguinal hernia is a rare occurrence and can have life-changing consequences if inadvertently damaged. It is unlikely to be diagnosed pre-operatively with the current approach to preparation for inguinal hernia repair. Patients with clinical or biochemical evidence of ureteric obstruction should be imaged pre-operatively; however, consideration for pre-operative imaging may also be considered for patients with known risk factors for ureteroinguinal hernia.
